# Sleep disorders in patients with myasthenia gravis based on antibody subtypes: a polysomnographic study

**DOI:** 10.1186/s12883-026-04881-x

**Published:** 2026-04-15

**Authors:** Beyza Arslan, Kayihan Uluc, Kadriye Agan, Pinar Kahraman Koytak, Gulin Sunter

**Affiliations:** 1 Department of Neurology & Clinical Neurophysiology, Van Yuzuncu Yil University Medical Faculty, Van, Turkey; 2 Department of Neurology & Clinical Neurophysiology, Marmara University Medical Faculty, Pendik, Istanbul, 34899 Turkey; 3 Neurology Clinic, Atasehir Acibadem Hospital, Istanbul, Turkey

**Keywords:** Myasthenia gravis, Sleep disorders, Obstructive sleep apnea syndrome, Muscle specific kinase antibody, Polysomnography, Multiple sleep latency test

## Abstract

**Background:**

Sleep-disordered breathing (SDB) is increasingly recognized in myasthenia gravis (MG), yet potential differences in sleep respiratory patterns between antibody-defined MG subtypes remain poorly understood. We aimed to evaluate sleep disorders and polysomnographic characteristics in patients with acetylcholine receptor antibody–positive (AChR-MG) and muscle-specific tyrosine kinase antibody–positive (MuSK-MG) generalized MG.

**Methods:**

Eighteen clinically stable MG patients (9 AChR-MG, 9 MuSK-MG) and 9 healthy controls underwent attended overnight polysomnography followed by the Multiple Sleep Latency Test (MSLT). Standardized sleep questionnaires and MG clinical scales were administered to assess sleep-related symptoms and disease severity.

**Results:**

Obstructive sleep apnea was detected in 89% of AChR-MG and 78% of MuSK-MG patients. Both MG subgroups showed significantly higher apnea–hypopnea index values compared with controls (*p* < 0.05). Although overall AHI severity did not differ significantly between antibody groups, distinct respiratory patterns were observed. Respiratory events clustered predominantly during REM sleep in AChR-MG, whereas MuSK-MG patients exhibited more frequent events during NREM or supine sleep.

**Conclusions:**

Sleep-disordered breathing appears to be common in MG, but polysomnographic patterns may differ according to antibody subtype. REM-predominant respiratory events in AChR-MG and NREM or positional predominance in MuSK-MG may reflect differences in muscle involvement between these subtypes. These findings suggest that antibody-specific mechanisms may influence nocturnal respiratory dysfunction in MG and highlight the importance of targeted sleep assessment in this population.

## Background

Myasthenia gravis (MG) is a chronic autoimmune disorder affecting the neuromuscular junction of skeletal muscle. It is characterized by fatigable muscle weakness. In MG, it has been reported that oropharyngeal, intercostal, and diaphragmatic muscle weakness, which becomes more pronounced during REM sleep, causes sleep disorders by creating hypoxemia [1]. As with many neuromuscular diseases, sleep-disordered breathing (SDB) in MG often appears earlier than daytime respiratory symptoms and is seen as a significant cause of morbidity and mortality [2]. 

An increased prevalence of sleep disorders has been observed in patients with MG; approximately 40–60% of clinically stable patients exhibit SDB [3]. Additionally, MG frequently coexists with other autoimmune conditions, raising interest in potential associations with autoimmune sleep disorders such as narcolepsy [4]. However, the prevalence and characteristics of sleep abnormalities in MG remain insufficiently documented. Early polysomnography-based studies by Manni et al. and Amino et al. demonstrated that respiratory disturbances during sleep can occur even in well-controlled MG, often manifesting as REM-predominant or mild central apneas [5, 6]. While these initial investigations provided important insights into sleep-related respiratory mechanisms in MG, they did not include objective assessment of daytime sleepiness or antibody-specific analyses. A recent systematic review also noted that most available studies relied solely on clinical questionnaires without incorporating overnight polysomnography (PSG), the gold standard for diagnosing sleep disorders [7]. Building upon these earlier findings, the present study combined PSG with the Multiple Sleep Latency Test (MSLT) to provide a comprehensive, antibody-specific evaluation of sleep disorders in MG.

In MG, the most frequently detected pathogenic autoantibodies target the acetylcholine receptor (AChR). In recent years, following the increase in knowledge about the components of the neuromuscular junction, new pathogenic antibodies such as muscle-specific tyrosine kinase (MuSK), low-density lipoprotein receptor–related protein 4 (LRP4), titin, and agrin have been identified. This has allowed for the disease to be classified into subgroups for clinical and therapeutic examination. Among these, MG characterized by MuSK antibodies is a rare disease, accounting for 5–8% of all patients with MG. It has distinct pathogenic and clinical features. It usually has an acute onset, predominantly affecting cranial, bulbar, and axial muscles, with frequent early respiratory crises. Diagnosis is often challenging due to the high frequency of atypical presentations, minimal fluctuation in clinical symptoms, frequent lack of response to acetylcholinesterase inhibitors, and the possibility of negative electrodiagnostic findings in limb muscles [8]. Because of these different clinical features, we aimed to examine sleep disorders in patients with MG separately in AChR-MG and MuSK-MG groups.

In the present study, we aimed to investigate polysomnographic characteristics of sleep disorders in clinically stable patients with AChR-MG and MuSK-MG. Using overnight polysomnography and the Multiple Sleep Latency Test, we specifically examined whether antibody-defined MG subtypes exhibit distinct patterns of sleep-disordered breathing. In addition, we evaluated the relationships between electrophysiological findings, clinical scales, and disease characteristics.

## Methods

### Study design and participants

This prospective, cross-sectional, observational study was conducted at the Marmara University Neuromuscular Diseases Clinic (Istanbul, Turkey) between 2021 and 2022. A total of 27 participants were included: nine patients with AChR-MG, nine with MuSK-MG, and nine healthy controls. Because the number of clinically stable MuSK-MG patients meeting the inclusion criteria during the study period was limited, this group represented the limiting subgroup for the study design. Therefore, the AChR-MG and control groups were selected in equal numbers to allow balanced exploratory comparisons between antibody-defined MG subtypes.

All MG patients had generalized myasthenia gravis and were clinically stable, defined as the absence of exacerbation or medication adjustment for at least 12 months prior to inclusion. Patients were consecutively recruited from individuals regularly followed in the neuromuscular disorders clinic who agreed to participate in the sleep evaluation protocol. All participants underwent polysomnographic evaluation based on the study protocol, regardless of pre-existing sleep complaints or clinical suspicion of sleep apnea.

Healthy controls were recruited among individuals without known neuromuscular or cardiopulmonary disease who volunteered for sleep laboratory evaluation. Polysomnographic studies in this group were performed for research purposes within the study protocol and were not limited to clinical indications of sleep disorders. Exclusion criteria for all participants included known cardiopulmonary diseases (e.g., chronic obstructive pulmonary disease, heart failure), other neuromuscular disorders, anatomical variations significantly affecting the upper airway (e.g., severe tonsillar hypertrophy), and alcohol or substance abuse, to ensure that the observed sleep-related respiratory patterns were primarily associated with MG.

Demographic characteristics including age, sex, neck circumference, height, weight, and body mass index (BMI) were recorded for all participants. Disease severity in MG patients was classified using the Myasthenia Gravis Foundation of America (MGFA) clinical classification [9]. 

### Sleep assessments

All participants underwent attended overnight polysomnography (PSG) followed by a Multiple Sleep Latency Test (MSLT) on the following day. Sleep studies were conducted as part of a structured research protocol and were not limited to patients with clinical suspicion of sleep disorders. These evaluations were conducted within the routine capacity of the sleep laboratory without external funding. The recording montage consisted of frontal, central and occipital electroencephalograms placed according to the international 10–20 system, a bipolar submental electromyogram, thoracic and abdominal inductance plethysmography, airflow via nasal-oral thermocouple, nasal pressure via nasal cannula, pulse oximetry (SpO₂ monitoring), electrocardiogram, body position monitoring via mercury gauge sensor, bilateral leg movements via piezoelectric sensors, right and left-sided electrooculograms. All recordings were manually scored by a certified sleep specialist according to standard criteria. The following parameters were obtained from PSG recordings: total recording time (TRT), total sleep time (TST), sleep latency (SL), REM-sleep latency (REML), sleep efficiency (SE), wake after sleep onset (WASO), duration and percentage of wakefulness and sleep stages (N1, N2, N3, and R), apnea-hypopnea index (AHI), AHI during NREM sleep stages (AHI-_NREMI_), AHI during REM stage (AHI-_REMI_), AHI in lateral position (AHI-_lateral_), AHI in supine position (AHI-_Supine_), mean oxygen saturation (MOS), and index of periodic leg movements in sleep (PLMSI). On the basis of these data, the diagnoses and severity of obstructive sleep apnea (OSA) were defined according to International Classification of Sleep Disorders [10]. The Multiple Sleep Latency Test was performed according to standard procedures to objectively evaluate daytime sleepiness and to exclude central hypersomnolence disorders such as narcolepsy. Because MG is an autoimmune disease and rare associations with narcolepsy type 1 have been reported, MSLT was included in the study protocol to investigate potential central hypersomnolence in this population. Sleep latency was defined as the time from lights off to the first epoch scored as sleep. A sleep-onset REM period (SOREM) was defined as the occurrence of REM sleep within 15 min after sleep onset [11]. 

#### Questionnaires

The following validated questionnaires were administered to assess sleep-related symptoms: the STOP-Bang questionnaire [12], the Berlin questionnaire [13], the Epworth Sleepiness Scale (ESS) [14], the Pittsburgh Sleep Quality Index (PSQI) [15], the Pittsburgh Insomnia Rating Scale (PIRS) [16] and the Insomnia Severity Index (ISI) [17]. The RLS questionnaire and severity scale were applied to AChR-MG and MuSK-MG groups to detect and evaluate the presence of sleep problems. RLS was diagnosed through diagnostic criteria established by a consensus of RLS experts from the International RLS Study Group in 2014 [18]. 

### Myasthenic clinical assessment

MG Activities of Daily Living (MG-ADL) [19], Quantitative MG (QMG) Score [20], MG Composite Scale (MGC) [21], MG Quality of Life 15 (MGQoL-15) [22] scales were applied for myasthenic clinical evaluation. All questionnaires and clinical scales used in this study are previously published and validated instruments.

### Data analysis

Statistical analyses were performed using SPSS version 26 (IBM Corp., Armonk, NY, USA). The distribution of continuous variables was assessed using descriptive statistics, graphical methods, and the Shapiro–Wilk test. Categorical variables were expressed as frequency and percentage, normally distributed continuous variables as mean ± standard deviation, and non-normally distributed variables as median (minimum–maximum). Comparisons between two groups were performed using the independent-samples t-test for normally distributed variables and the Mann–Whitney U test for non-normally distributed variables. Comparisons among more than two groups were conducted using one-way ANOVA or the Kruskal–Wallis test as appropriate. Correlations between continuous variables were analyzed using Pearson’s correlation test for normally distributed data and Spearman’s correlation test for non-normally distributed data. A p-value of < 0.05 was considered statistically significant.

The study protocol was approved by the Research Ethics Committee of Marmara University, Istanbul, Turkey (approval number: 1289/2022). Written informed consent was obtained from all participants.

## Results

### Demographic characteristics

A total of 27 participants were included: 9 patients with AChR-MG, 9 patients with MuSK-MG, and 9 healthy controls. Demographic characteristics of the study population are summarized in Table 1. The three groups were comparable in terms of age, sex distribution, body mass index, and neck circumference.

**Table 1:** Demographic and clinical characteristics

	AChR-MG (*n* = 9)	MuSK-MG (*n* = 9)	Control (*n* = 9)	*p*-value
Age (years)	38.56 ± 11.01	40.56 ± 10.10	38.44 ± 14.18	0.914^a^
Female (n, %)	7 (77.8%)	7 (77.8%)	6 (66.7%)	0.999^b^
BMI (kg/m²)	28.01 ± 5.45	27.83 ± 4.23	27.38 ± 3.97	0.956
Neck Circumference (cm)	36.79 ± 3.24	37.33 ± 4.44	37.22 ± 4.15	0.954
MGFA Classification	I (5), IIa (4)	I (4), IIa (5)	-	-
MG-ADL Score	1.22 ± 1.30	1.44 ± 1.51	-	0.742
QMG Score	7.33 ± 3.74	4.78 ± 4.27	-	0.195
MGC Score	2.11 ± 2.52	3.89 ± 3.41	-	0.226
MGQoL-15 Score	13.00 ± 7.92	5.22 ± 4.44	-	0.024^c*^

*AChR-MG* Acetylcholine receptor antibody-positive myasthenia gravis,*MuSK-MG* Muscle-specific kinase antibody-positive myasthenia gravis,*BMI* Body mass index,*MGFA* Myasthenia Gravis Foundation of America*MG-ADL* Myasthenia Gravis Activities of Daily Living,*QMG* Quantitative Myasthenia Gravis,*MGC* Myasthenia Gravis Composite,*MGQoL-15* Myasthenia Gravis Quality of Life-15

*p*> 0.05

^a^: One-way ANOVA test, ^b^: Fisher’s exact test, **#**: mean±standard deviation, c: Independent samples test, *: Dunn’s multipl comparison test

All patients with MG were classified as MGFA class I or IIa and were clinically stable at the time of evaluation. The mean age was 38.56 ± 11.01 years in the AChR-MG group and 40.56 ± 10.10 years in the MuSK-MG group. Female participants predominated in all groups (78% in AChR-MG, 78% in MuSK-MG, and 67% in controls).

Within the AChR-MG group, age showed a positive correlation with AHI-NREM (*r* = 0.476, *p* = 0.046) and AHI-lateral (*r* = 0.469, *p* = 0.049). In the MuSK-MG group, neck circumference correlated positively with AHI (*r* = 0.714, *p* = 0.031), AHI-NREM (*r* = 0.756, *p* = 0.018), and AHI-supine (*r* = 0.740, *p* = 0.023).

### Polysomnographic findings

Polysomnographic findings are summarized in Table 2.

**Table 2:** Polysomnography (PSG) and Mean Sleep Latency Test (MSLT) findings

	AChR-MG (*n* = 9)	MuSK-MG (*n* = 9)	Control (*n* = 9)	*p*-value
Diagnosis of OSA (%)	8 (88.9%)	7 (77.8%)	3 (33.3%)	0.002
AHI (events/hour)	6.9(3.3–34.8)	8.4(1.5–24.4)	3.7(0.0-4.9)	0.003
AHI-_NREMI_	6.1(1.2–35.4)	6.6(1.4–24.2)	2.5(0.0-4.8)	0.005
AHI-_REMI_	11.8(0.0–38.0)	13.0(0.0-30.7)	5.1(0.0-21.4)	0.003
AHI-_Lateral_	6.1(0.00-34.80)	4.8(1.00–24.00)	0.0(0.00–4.00)	0.006
AHI_−supine_	6.2(0.0-46.3)	16.3(3.1–45.2)	5.7(0.0-8.1)	0.153
Mean Oxygen Saturation (%)	95.2(93.2–97.0)	95.9(91.5–97.4)	96.3(92.9–98.2)	0.390^b^
Sleep Efficiency (%)	78.78 ± 18.82	80.99 ± 14.65	86.27 ± 7.90	0.540^a^
WASO	57.5(8.6-218.7)	59.8(11.5-172.4)	29.3(9.4–91.6)	0.623^b^
PLMSI	0.0(0.0–0.0)	0.0(0.0-6.7)	0.0(0.0-48.5)	0.167^b^
MSLT-_sleep latancy_	11.1(2.8–17.6)	12.9(3.6–15.5)	13.1(9.5–17)	0.383^b^
Number of SOREMs	0.0(0.0–0.0)	0.0(0.0–0.0)	0.0(0.0–0.0)	-

Data are presented as mean ± standard deviation for normally distributed variables and median (range) for non-normally distributed variables; categorical variables are shown as number (percentage)

*Osa* obstructive sleep apnea, *ahi* apnea–hypopnea index, *rem* rapid eye movement, *nrem* non-rapid eye movement, *plmsi* periodic limb movement sleep index, *waso* wake after sleep onset *mslt* multiple sleep latency test, *sorem* sleep onset rem period

*p*-values were calculated using independent-sample t-test or Mann–Whitney U test, as appropriate

^a^: One-way ANOVA test ^b^: Kruskal-wallis-H test

Obstructive sleep apnea (OSA) was diagnosed in 89% of patients with AChR-MG and 78% of those with MuSK-MG, compared with 34% of the control group.

Although overall AHI values did not differ significantly between the AChR-MG and MuSK-MG groups, distinct patterns of respiratory events were observed between the antibody-defined subtypes. In the AChR-MG group, respiratory events tended to occur predominantly during REM sleep, reflected by relatively higher AHI-REM values. In contrast, patients with MuSK-MG demonstrated relatively higher respiratory events during NREM sleep and in the supine position.

Compared with controls, both MG groups had significantly higher AHI, AHI-NREM, AHI-REM, and AHI-lateral values (*p* < 0.05). Higher AHI-supine values were observed in the MuSK-MG group relative to the other groups, although this difference did not reach statistical significance.

Sleep efficiency tended to be lower and wake after sleep onset higher in both MG groups compared with controls, although these differences were not statistically significant. Consistent with the predominantly mild-to-moderate severity of OSA in our cohort, mean oxygen saturation (MOS) and minimum oxygen saturation values remained within similar ranges across all groups, with no significant differences observed (Table 2).

### Daytime sleepiness and sleep-related symptoms

No significant differences in mean sleep latency on the Multiple Sleep Latency Test were observed between the groups. However, patients with AChR-MG showed shorter sleep latencies and higher Epworth Sleepiness Scale scores compared with the MuSK-MG and control groups. No sleep-onset REM periods (SOREM) were observed in any participant.

Periodic limb movement index values were within normal limits in all participants, and none of the patients fulfilled diagnostic criteria for restless legs syndrome.

No significant differences were found between the AChR-MG and MuSK-MG groups in sleep-related questionnaire scores.

### Clinical correlations

In the MuSK-MG group, Berlin questionnaire scores correlated positively with AHI (*r* = 0.671, *p* < 0.05), AHI-NREM (*r* = 0.671, *p* < 0.05), AHI-supine (*r* = 0.689, *p* < 0.05), and AHI-lateral (*r* = 0.820, *p* < 0.01). In contrast, no significant correlations between these parameters were observed in the AChR-MG group.

A positive correlation was observed between MG-ADL scores and AHI-REM values in the AChR-MG group (*r* = 0.762, *p* < 0.05). In the MuSK-MG group, QMG scores correlated positively with PSQI (*r* = 0.701, *p* < 0.05), PIRS (*r* = 0.733, *p* < 0.05), and ISI (*r* = 0.760, *p* < 0.05).

## Discussion

This study evaluated sleep disorders in clinically stable patients with antibody-defined myasthenia gravis using overnight polysomnography and the Multiple Sleep Latency Test. Our findings demonstrate that sleep-disordered breathing is highly prevalent in MG even in the absence of clinical exacerbation. However, the reported prevalence rates should be interpreted with caution given the limited sample size and potential selection bias inherent to the study design. More importantly, distinct polysomnographic patterns were observed between antibody-defined MG subtypes.

### Sleep-disordered breathing patterns in MG subtypes

The prevalence of OSA in our cohort (83%) is consistent with previous studies reporting increased rates of sleep-disordered breathing in patients with myasthenia gravis [3, 23]. For example, Nicolle et al. demonstrated that obstructive sleep apnea occurs more frequently in MG patients than in the general population based on polysomnographic evaluation [24]. However, most earlier investigations primarily focused on the prevalence of OSA rather than examining differences in respiratory event patterns between MG subtypes [25]. 

Both AChR-MG and MuSK-MG patients in our study exhibited significantly higher AHI values compared with controls, although overall OSA severity did not differ significantly between the two antibody-defined subgroups. Despite similar overall AHI values, our polysomnographic analysis revealed different patterns of respiratory events. Patients with AChR-MG tended to experience respiratory events predominantly during REM sleep, whereas MuSK-MG patients demonstrated relatively more frequent respiratory events during NREM sleep and in the supine position.

These findings may reflect the distinct pathophysiological mechanisms underlying antibody-defined MG subtypes. AChR-MG is typically associated with fluctuating oropharyngeal and diaphragmatic weakness, which becomes more pronounced during REM sleep because of physiological muscle atonia. In contrast, MuSK-MG is characterized by more persistent bulbar and axial muscle involvement, which may predispose patients to position-related or non-REM-related respiratory instability.

Our findings are also consistent with earlier observations by Manni et al. and Amino et al., who demonstrated that respiratory disturbances during sleep can occur in MG even in clinically stable patients without overt daytime respiratory symptoms [5, 6]. In our cohort, MG-ADL scores correlated with AHI-REM values in the AChR-MG group, while neck circumference correlated with positional AHI indices in the MuSK-MG group, suggesting that both disease-specific muscle involvement and anthropometric parameters contribute to nocturnal respiratory dysfunction.

Importantly, unlike earlier studies such as that by Nicolle et al., which primarily evaluated the prevalence of obstructive sleep apnea in MG populations, our study focused on the polysomnographic characteristics of respiratory events across antibody-defined MG subtypes. This approach allowed identification of distinct REM-predominant and position-related respiratory patterns associated with different MG phenotypes.

### Clinical implications

Although most OSA cases in our cohort were mild or moderate, five patients (three AChR-MG and two MuSK-MG) met AASM criteria for CPAP therapy and reported improvement in daytime alertness after treatment [26]. These findings support previous reports indicating that nocturnal hypoxemia and sleep fragmentation may exacerbate neuromuscular fatigue and negatively affect quality of life in MG.^5^ The absence of significant nocturnal desaturation, as evidenced by stable mean oxygen saturation levels despite a high SDB prevalence, further reflects the clinical stability of our study population and suggests that respiratory events in these patients do not typically lead to severe hypoxemia.

Importantly, several patients diagnosed with OSA did not report prominent sleep-related complaints. This observation highlights the potential value of systematic sleep evaluation in MG populations, even among clinically stable patients.

The identification of different respiratory patterns between MG subtypes may also have practical implications. Particularly for MuSK-MG patients, positional factors appeared highly relevant; therefore, positional therapy -such as side-sleeping or using a wedge to place the diaphragm in a more advantageous position- could effectively complement conventional OSA management in this subgroup. Furthermore, in patients with prominent bulbar symptoms, optimizing sleep posture with specialized pillows to avoid chin tuck -which reduces the caliber of the upper airways- or the use of oral appliances to address tongue weakness may offer significant clinical benefits beyond standard CPAP therapy.

Given the high prevalence of sleep-disordered breathing observed in this cohort, simplified respiratory-focused sleep studies may be considered as an initial screening approach in selected MG patients. However, our study highlights that full polysomnography remains essential for a comprehensive evaluation, as it enables the detection of nuanced, stage-specific respiratory patterns that may be missed by simplified screening methods, thus providing a more detailed phenotyping of antibody-defined MG subgroups.

### Screening and diagnostic considerations

In our study, a positive correlation was observed between Berlin questionnaire scores and AHI indices in the MuSK-MG group, suggesting that validated screening tools may help identify patients at increased risk of sleep-disordered breathing who warrant further evaluation with polysomnography.

Previous studies have shown that the diagnostic performance of screening questionnaires such as STOP-Bang and Berlin may vary in populations with comorbid medical conditions [27–29]. Our findings suggest that the Berlin questionnaire may have greater predictive value than STOP-Bang for detecting OSA risk in patients with MuSK-MG. Furthermore, the lack of correlation in certain subgroups might suggest that sleep-disordered breathing in MG is primarily driven by specific neuromuscular factors -such as bulbar or diaphragmatic weakness- which may not be adequately captured by traditional OSA screening tools that heavily weight factors like obesity and neck circumference. In addition, the relatively small sample size and the predominance of mild OSA in this cohort may have reduced the sensitivity of questionnaire-based screening tools to detect objective respiratory abnormalities, particularly in the AChR-MG subgroup, where correlations with AHI were not observed. Furthermore, OSA was identified in several MG patients without overt sleep complaints, emphasizing the importance of routinely evaluating sleep-related symptoms in this population.

### Daytime sleepiness and central hypersomnolence

Although patients with MG frequently report excessive daytime sleepiness, objective investigations of hypersomnolence in this population remain limited [7, 30]. In the present study, MSLT results did not demonstrate evidence of central hypersomnolence disorders. No sleep-onset REM periods were observed, and mean sleep latency values were comparable between groups.

These findings suggest that daytime sleepiness in MG is more likely related to sleep fragmentation and nocturnal respiratory disturbances rather than primary hypersomnolence disorders such as narcolepsy or idiopathic hypersomnia. This observation is noteworthy given the proposed autoimmune links between MG and narcolepsy type 1, although such associations appear to be rare [4]. We acknowledge that specific narcolepsy screening instruments, such as the Ullanlinna Narcolepsy Scale or the Narcolepsy Severity Scale, were not applied in this study [31, 32]. However, no clinical features suggestive of narcolepsy were identified during patient evaluation, and MSLT findings did not support a diagnosis of central hypersomnolence.

### Insomnia and sleep quality

No significant associations were observed between PSG parameters and MGQoL-15 scores in our cohort, possibly reflecting the predominance of mild OSA severity. However, in MuSK-MG patients, QMG scores correlated with insomnia-related scales (PIRS, ISI, and PSQI), suggesting that disease severity may contribute to subjective sleep complaints in this subgroup.

Previous studies have proposed several factors that may contribute to insomnia in MG, including age, thymectomy status, corticosteroid exposure, and respiratory symptoms [33]. In our cohort, MuSK-MG patients were clinically stable and not receiving corticosteroid therapy, raising the possibility that disease-specific neuromuscular involvement may contribute to sleep maintenance difficulties [34]. 

### Restless legs syndrome and periodic limb movements

Although previous studies have reported increased prevalence of restless legs syndrome in MG populations, none of the patients in our cohort fulfilled diagnostic criteria for RLS, and PLMS indices remained within normal limits [35]. While the relatively small sample size may limit detection of less frequent comorbidities, these findings suggest that RLS and PLMD are unlikely to represent major contributors to sleep disturbances in clinically stable MG populations.

### Study limitations

This study has several limitations. First, the relatively small sample size and single-center design may limit the generalizability of the findings. In addition, participants were recruited among patients regularly followed in a neuromuscular clinic who agreed to undergo polysomnographic evaluation, which may introduce a degree of selection bias and may have contributed to an overestimation of sleep-disordered breathing prevalence. However, the number of eligible patients during the study period was inherently limited because only clinically stable individuals meeting strict inclusion criteria and willing to participate in the sleep laboratory protocol could be included. Furthermore, MuSK-MG is a relatively rare subtype of myasthenia gravis, and only nine clinically stable MuSK-MG patients met the inclusion criteria during the study period. Therefore, the comparison groups were selected in equal numbers to allow balanced exploratory comparisons between antibody-defined subtypes. Finally, the use of a single-night PSG may not fully capture night-to-night variability in sleep-disordered breathing severity. In addition, specific narcolepsy screening scales were not applied, which may limit the evaluation of subtle clinical features of central hypersomnolence, although no clinical or electrophysiological evidence of central hypersomnolence was identified.

## Conclusions

Sleep-disordered breathing appears to be common in clinically stable myasthenia gravis. Although overall OSA severity appears similar across antibody-defined subtypes, our findings suggest that respiratory event patterns may differ between AChR-MG and MuSK-MG. REM-predominant respiratory events were more common in AChR-MG, whereas MuSK-MG patients demonstrated relatively more NREM- or position-related respiratory events.

These observations suggest that antibody-specific neuromuscular involvement may influence nocturnal respiratory physiology in MG. Recognizing these patterns may help guide more individualized sleep evaluation and management strategies in patients with myasthenia gravis.

## Data Availability

The datasets used and/or analyzed during the current study are available from the corresponding author on reasonable request.
